# Aortic Oxidative Stress, Inflammation and DNA Damage Following Pulmonary Exposure to Cerium Oxide Nanoparticles in a Rat Model of Vascular Injury

**DOI:** 10.3390/biom9080376

**Published:** 2019-08-17

**Authors:** Abderrahim Nemmar, Suhail Al-Salam, Sumaya Beegam, Priya Yuvaraju, Badreldin H. Ali

**Affiliations:** 1 Department of Physiology, College of Medicine and Health Sciences, United Arab Emirates University, P.O. Box 17666, Al Ain, Abu Dhabi, UAE; 2 Department of Pharmacology, College of Medicine & Health Sciences, Sultan Qaboos University, P.O. Box 35, Muscat 123, Al-Khoud, Oman

**Keywords:** Cerium oxide nanoparticles, aorta, inflammation, oxidative stress, Nrf2, DNA damage

## Abstract

Pulmonary exposure to cerium oxide nanoparticles (CeO_2_ NPs) can occur either at the workplace, or due to their release in the environment. Inhaled CeO_2_ NPs are known to cross the alveolar–capillary barrier and reach various parts of the body, including the vasculature. The anticancer drug cisplatin (CP) causes vascular damage. However, the effects CeO_2_ NPs on vascular homeostasis in a rat model of CP-induced vascular injury remain unclear. Here, we assessed the impact and underlying mechanism of pulmonary exposure to CeO_2_ NPs on aorta in rats given a single intraperitoneal injection of cisplatin (CP, 6 mg/kg) to induce vascular damage. Six days later, the rats were intratracheally instilled with either CeO_2_ NPs (1 mg/kg) or saline (control), and various variables were studied 24 h thereafter in the aortic tissue. The concentration of reduced glutathione and the activity of catalase were significantly increased in the CP + CeO_2_ NPs group compared with both the CP + saline and the CeO_2_ NPs groups. The activity of superoxide dismutase was significantly decreased in the CP + CeO_2_ NPs group compared with both the CP + saline and CeO_2_ NPs groups. The expression of nuclear factor erythroid-derived 2-like 2 (Nrf2) by the nuclei of smooth muscles and endocardial cells assessed by immunohistochemistry was significantly augmented in CeO_2_ NPs versus saline, in CP + saline versus saline, and in CP + CeO_2_ NPs versus CeO_2_ NPs. Moreover, the concentrations of total nitric oxide, lipid peroxidation and 8-hydroxy-2-deoxyguanosine were significantly elevated in the CP + CeO_2_ NPs group compared with both the CP + saline and the CeO_2_ NPs groups. Similarly, compared with both the CP + saline and CeO_2_ NPs groups, the combination of CP and CeO_2_ NPs significantly elevated the concentrations of interleukin-6 and tumour necrosis factor-α. Additionally, aortic DNA damage assessed by Comet assay was significantly increased in CeO_2_ NPs compared with saline, and in CP + saline versus saline, and all these effects were significantly aggravated by the combination of CP and CeO_2_ NPs. We conclude that pulmonary exposure to CeO_2_ NPs aggravates vascular toxicity in animal model of vascular injury through mechanisms involving oxidative stress, Nrf2 expression, inflammation and DNA damage.

## 1. Introduction

Nanotechnology develops particles < 100 nm in size in at least one dimension with unique physical and chemical characteristics. These nanoparticles are being used in many fields, including industrial, medical, information, and communication technologies, implying wide use in consumer and industrial products [1]. However, the manufacturing of novel nanomaterials frequently occurs in the absence of relevant studies on the possible pathophysiological effects on human health [2,3,4].

Among the various nanomaterial products, cerium oxide (CeO_2_) is an important compound, as it is used in solar and fuel cells, gas sensors, oxygen pumps, polishing media, and as a fuel additive to decrease emanations of gaseous products of incomplete combustion, and enhance fuel burning efficiency [5,6,7]. In addition to human exposure in the workplace during manufacturing, it has been reported that CeO_2_ nanoparticles (CeO_2_ NPs) are discharged into ambient air from diesel engines that use cerium compounds as a diesel fuel catalyst, leading to exposure of humans by inhalation [5,6,7].

In addition to causing lung toxicity, inhaled CeO_2_ NPs have been shown to induce secondary systemic effects by their ability to pass through the alveolar-capillary barrier and/or CeO_2_ NPs-induced release of reactive oxygen species and inflammatory mediators from the alveoli into the circulation, affecting various distant sites, including the vascular tissue [8,9]. Moreover, human and animal investigations have established that the pathophysiological effects of nanoparticles in general are aggravated in vulnerable patients and animals with pre-existing vascular diseases [3,10,11]. However, as far as we are aware, the impact of pulmonary exposure to CeO_2_ NPs on the aorta in an animal model of vascular injury induced by cisplatin (CP) has not been reported before. CP is an effective anticancer drug utilized in the treatment of several solid tumours [12,13,14]. However, its usage is associated with many severe adverse effects, particularly nephrotoxicity and vascular dysfunction [12,13,14]. Even though the mechanisms of action of CP are not fully understood, several studies have reported that CP-induced cytotoxicity involves oxidative stress and inflammation, and causes vascular toxicity including myocardial infarction and stroke [12,13,14]. Vascular dysfunction can progress into systemic vascular injury, which is principally classified into macrovascular disease comprising aortic atherosclerosis and microangiopathy. In the present study, the rat aorta was used to investigate the pathophysiological alterations at macrovascular level. The aorta was also chosen because such large vessels can be easily collected, making it possible to assess the possible aggravating effects of the combination of pulmonary exposure to CeO_2_ NPs and CP treatment on aortic oxidative stress, inflammation and DNA damage. The latter events are observed in the early stage of aortic atherosclerosis [12,13,14].

Therefore, the aim of this report is to assess the impact and mechanism of lung exposure to CeO_2_ NPs on the aorta of healthy rats, and rats with CP-induced vascular damage by measuring several markers of inflammation (interleukin-6 (IL-6) and tumour neurosis factor-α (TNF α)), oxidative stress (reduced glutathione (GSH), catalase (CAT), superoxide dismutase (SOD), nuclear factor erythroid-derived 2-like 2 (Nrf2) expression, total nitric oxide (NO), lipid peroxidation (LPO) and 8-hydroxydeoxyguanosine (8-OHdG)) and DNA damage. To our knowledge, such an interaction has never been reported before.

## 2. Material and Methods

### 2.1. Particles

CeO_2_ NPs, 10 wt. % in water with an average diameter of ~20 nm, were obtained from Sigma-Aldrich (St Louis, MO, USA). CeO_2_ NPs samples diluted in saline were used for rat exposures. To minimize aggregation, particle suspensions were always sonicated for 5 min (Clifton Ultrasonic Bath, Clifton, NJ, USA). Particle suspensions were prepared promptly before use and were vortexed to obtain well mixed suspension prior to each instillation. The same particles from the same source were characterized and used recently by Ma et al. and by us [8,15,16,17].

The endotoxin concentration in the CeO_2_ NPs and saline used was quantified, as described by the manufacturer, by chromogenic Limulus Amebocyte Lysate (Pierce, Rockford, IL, USA) test. The concentrations were lower than the detection limit (0.1 EU/mL) in the saline and CeO_2_ NPs solutions.

### 2.2. Animals and i.t. Instillation

This project was reviewed and approved by the Institutional Review Board of the United Arab Emirates University (ERA_2016_4408, 17/11/2016), and experiments were performed in accordance with protocols approved by the Institutional Animal Care and Research Advisory Committee.

A total number of 76 male Wistar rats (Taconic Farms Inc., Germantown, New York, USA) were used in this study. Rats were aged 10–12 weeks and, initially weighing 223 ± 13 g, were given a standard laboratory chow and water ad libitum. They were randomly divided into four groups and individually housed at a temperature of 23 ± 2 °C, relative humidity of 50–60% and a 12 h dark-light cycle. The rats were weighed at the beginning of the experiment and just before sacrifice. Rats were cared for under a protocol approved by the Animal Research Ethics Committee of our college, and according to the NIH Guide for the Care and Use of Laboratory Animals, NIH publication no. 85-23, 1985.

### 2.3. Treatments

Vascular toxicity in rats was induced by a single intraperitoneal (i.p.) injection of CP (David Bull Laboratories, PTY Ltd., Victoria, Australia) at a dose of 6 mg/kg [18,19]. Control animals received a similar volume of normal saline i.p. On day 6 of treatment, the animals were anaesthetized by isoflurane inhalation, and placed supine with extended neck on an angled board. A Becton Dickinson 18 Gauge cannula was inserted via the mouth into the trachea. CeO_2_ NPs suspension (150 µL) or saline-only were instilled (150 µL) via a sterile syringe and followed by an air bolus of 100 µL. The dose of the CeO_2_ NPs was selected based on previous work [17].

The four groups were treated as follows:

**Group 1**: single normal saline (control, 500 μL/rat) given i.p., and on day 6 of the treatment, a single i.t. administration of saline (150 μL per rat);

**Group 2**: single normal saline (control, 500 μL/rat) given i.p., and on day 6 of the treatment, a single i.t. administration of CeO_2_ NPs (1 mg/kg);

**Group 3**: single CP (6 mg/kg) given i.p., and on day 6 of the treatment, a single i.t. administration of saline (150 μL per rat);

**Group 4**: single CP (6 mg/kg) given i.p., and on day 6 of the treatment, a single i.t. administration of CeO_2_ NPs (1 mg/kg);

Twenty-four hours after the i.t. instillation of CeO_2_ NPs or saline with or without CP treatment, various biochemical and histological endpoints were measured in the aortae of rats.

### 2.4. Sample Collection and Biochemical Analysis

For the biochemical analysis a sample size of 32 rats was used. The animals were sacrificed with an overdose of anaesthesia. The chest was opened and the thoracic aorta (arch to bifurcation) was quickly removed and kept in a 4 °C PBS (pH 7.4), and blood, connective tissue, and fat were removed from each vessel, and the aorta was cut into rings of 3–4 mm in length, weighed and subjected to homogenization for biochemical studies [8,20,21].

The aortic homogenates were prepared as described previously [8,21,22]. Homogenates were centrifuged for 10 min at 3000× *g* to remove cellular debris, and the supernatants were used for further analysis [21]. Protein content was measured by Bradford’s method. The NADPH-dependent membrane LPO was determined using a kit that measures thiobarbituric acid reactive substances (Cayman Chemical Company, Ann Arbor, MI, USA). GSH was measured with a kit obtained from Sigma-Aldrich Co (St Louis, MO, USA). The measurement of CAT and SOD activities was performed using kits from Cayman Chemical Company (Ann Arbor, MI, USA). The determination of NO was achieved with a total NO assay kit from R&D systems (Minneapolis, MN, USA) which measures the more stable NO metabolites NO_2_^−^ and NO_3_^−^ [23]. The aortic homogenate concentration of 8-OH-dG was quantified using an ELISA kit (Cayman, Ann Arbor, MI, USA) according to the manufacturer’s instructions. The concentrations of TNFα and IL-6 were determined using commercial Kits (Duo Set, R & D systems, Minneapolis, MN, USA). The number of animals per group was 8. Thus, for the majority of the biochemical parameters measured, we used *n* = 8, and in a few of them we used *n* = 5–7 (see figure legends). This was due to the volume of homogenate recovered from aortic tissues which was slightly variable. The latter depended on the animal weight and the weight of the aortic tissues collected. We were also limited by the amount of reagents available for some biochemical tests (total NO and 8-OH-dG), which allowed us to run *n* = 5–6.

### 2.5. Histology and Immunohistochemistry

For histological and immunohistochemistry analysis, a sample size of 24 rats (*n* = 6 per group) was used. Segments of aorta collected as described above were cassetted and fixed directly in 10% neutral formalin for 24 h, which was followed by dehydration in increasing concentrations of ethanol, clearing with xylene and embedding with paraffin. Three-μm sections were prepared from paraffin blocks and stained with haematoxylin and eosin. The stained sections were evaluated by the histopathologist (S.A.), using light microscopy.

Regarding immunohistochemistry, five-µm sections were cut, de-waxed with xylene and rehydrated with graded alcohol. The slides were then placed in a 0.01 M citrate buffer solution (pH = 6.0) and pre-treatment procedures to unmask the antigens were performed in a water bath for 60 min. Sections were treated with peroxidase and protein block for 15 min each and then incubated with the primary antibodies anti-Nrf2 (rabbit polyclonal antibody, Abcam, Cambridge, UK) for one hour at room temperature. After conjugation with primary antibody, sections were washed and then incubated with Dako REAL™ EnVision™/HRP for 1 h at room temperature (DAKO, Agilent, CA, USA), followed by washing and addition of DAB chromogen (DAKO, Agilent, Santa Clara, CA, USA). Sections were then counter stained with haematoxylin. Appropriate positive controls were used. For the negative control, the primary antibody was not added to sections and the whole procedure was carried out in the same manner as mentioned above. The immunohistochemical staining for Nrf2 was scored semi-quantitatively and blindly by our histopathologist on a scale of 0–4 according to the percentage of staining in 4 slides of each specimen, and each slide contained 4 equal coronal segments of the aorta. A score of 0 was assigned if the expression was 0–10%, 1 for 11–25%, 2 for 26–50%, 3 for 51–75% and 4 for more than 75% [24,25]. The number of nuclei of smooth muscles and endocardial cells stained with Nrf2 were counted at high power field (×100) [26].

### 2.6. Assessment of DNA Damage by COMET Assay

In separate sets of animals (*n* = 20, i.e., *n* = 5 per group), immediately after sacrifice, the aortae were removed from each animal as described above. Single-cell suspensions of the different aortae were obtained and analysed according to the method described in our previous publications [27,28,29,30]. Each aorta was washed in a chilled medium (RPMI 1640, 15% DMSO, 1.8% (*w*/*v*) NaCl). The aortic tissues were put in 1.5 mL medium and cut finely into pieces in a Petri dish. The slices were allowed to deposit, and the supernatant was collected in a 15 mL tube. The collected cell suspension was centrifuged at 1000 rpm for 5 min at 4 °C. The supernatant was removed, and the pellets were suspended in 0.5 mL of the medium. The rest of the procedure performed was as described earlier [27,28,29,30].

### 2.7. Statistics

All data were analysed with GraphPad Prism Version 4.01 for Windows software (Graphpad Software Inc., San Diego, CA, USA). Data were analysed for normal distribution using the D’Agostino and Pearson omnibus normality test. Data are expressed as means ± SEM. Comparisons between groups were performed by one-way analysis of variance (ANOVA), followed by Newman Keuls test for comparing treated with control groups. P values < 0.05 are considered significant.

## 3. Results

### 3.1. GSH Concentration and CAT and SOD Activities in Aortic Homogenates

**Figure 1:**
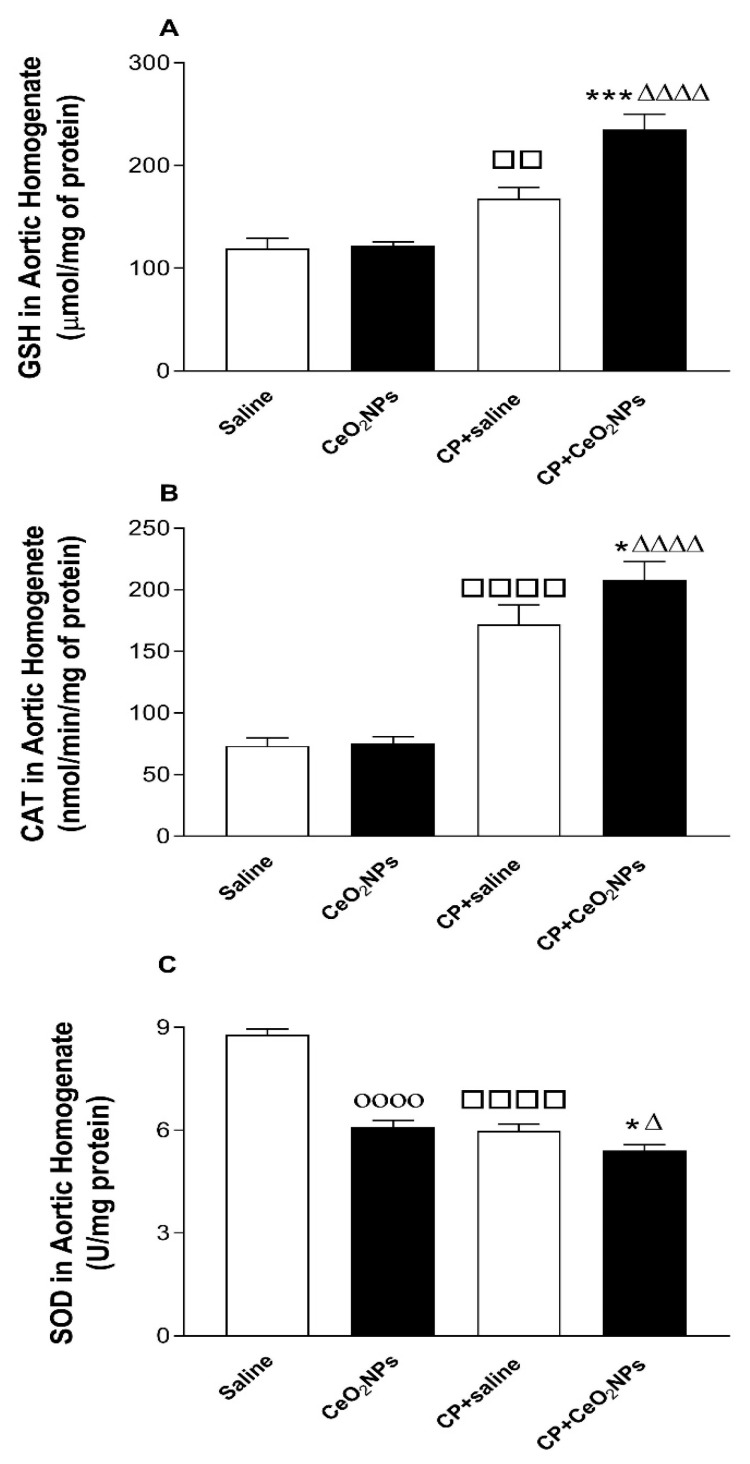
Glutathione (GSH, (**A**)) concentration in aortic homogenates of rats intratracheally instilled with either saline (*n* = 8) or CeO_2_ NPs (*n* = 8) with (*n* = 8) or without (*n* = 8) CP administration. *** *P* < 0.001 compared with CP + saline-treated group. ^ΔΔΔΔ^ *P* < 0.0001 compared with CeO_2_ NPs-treated group. ^□□^ *P* < 0.01 compared with saline-treated group. Catalase (CAT, (**B**)) activity in aortic homogenates of rats intratracheally instilled with either saline (*n* = 8) or CeO_2_ NPs (*n* = 8) with (*n* = 6) or without (*n* = 6) CP administration. * *P* < 0.05 compared with CP + saline-treated group. ^ΔΔΔΔ^ *P* < 0.0001 compared with CeO_2_ NPs-treated group. ^□□□□^ *P* < 0.0001 compared with the saline-treated group. Superoxide dismutase (SOD, (**C**)) activity in aortic homogenates of rats intratracheally instilled with either saline (*n* = 8) or CeO_2_ NPs (*n* = 8) with (*n* = 8) or without (*n* = 8) CP administration. * *P* < 0.05 compared with the CP + saline-treated group. ^Δ^ *P* < 0.05 compared with the CeO_2_ NPs-treated group. ^□□□□^ *P* < 0.0001 compared with the saline-treated group. ^οοοο^ *P* < 0.0001 compared with the saline-treated group. Data are mean ± SEM.

The quantification of the concentration of the free radical scavenger GSH and the activities of the antioxidant enzymes SOD, and CAT following pulmonary exposure to either saline or CeO_2_ NPs with or without CP administration are shown in Figure 1. Figure 1A shows that the concentration of GSH was significantly increased in CP + saline versus saline group (*P* < 0.01), and in CP + CeO_2_ NPs compared with either with either CP + saline (*P* < 0.001) or CeO_2_ NPs (*P* < 0.0001) group. Figure 1B displays that the activity of CAT was significantly increased in CP + saline versus saline group (*P* < 0.0001), and in CP + CeO_2_ NPs compared with either with either CP + saline (*P* < 0.05) or CeO_2_ NPs (*P* < 0.0001) group. Furthermore, Figure 1C shows that the activity of SOD was significantly decreased in CP + saline versus saline group (*P* < 0.0001), in CeO_2_ NPs versus saline group (*P* < 0.0001), and in CP + CeO_2_ NPs compared with either with either CP + saline (*P* < 0.05) or CeO_2_ NPs (*P* < 0.05) group.

### 3.2. Histopathological Analysis of the Aorta and Expression of Nrf2

**Figure 2:**
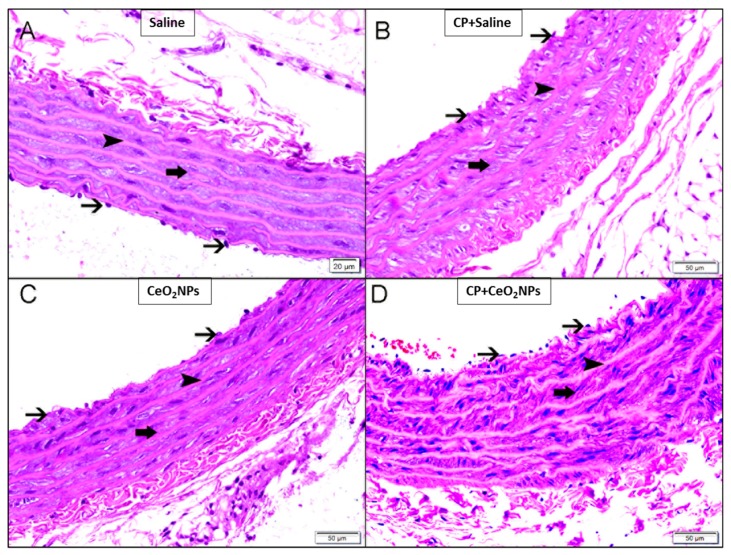
(**A**–**D**) Representative light microscopy sections of H and E-stained aortic tissues of rats intratracheally instilled with either saline (control) or cerium oxide nanoparticles (CeO_2_ NPs) with or without cisplatin (CP) administration. (**A**) Representative aortic section obtained from saline-treated rats displaying normal aorta with unremarkable changes showing endocardial cells lining (thin arrow), elastic fibres (arrowhead) and smooth muscles (thick arrow). (**B**) Representative aortic section obtained from rats treated with CP and intratracheally instilled with saline with unremarkable changes showing endocardial cells lining (thin arrow), elastic fibres (arrowhead) and smooth muscles (thick arrow). (**C**) Representative aortic section obtained from CeO_2_ NPs-treated rats displaying normal aorta with unremarkable changes showing endocardial cells lining (thin arrow), elastic fibres (arrowhead) and smooth muscles (thick arrow). (**D**) Representative aortic section obtained from rats treated with CP and intratracheally instilled with CeO_2_ NPs with unremarkable changes showing endocardial cells lining (thin arrow), elastic fibres (arrowhead) and smooth muscles (thick arrow).

Figure 2 suggests that there were no significant light–microscopic differences in the H&E-stained aortic sections between the four groups studied (saline, CeO_2_ NPs, CP + saline and CP + CeO_2_ NPs groups).

**Figure 3:**
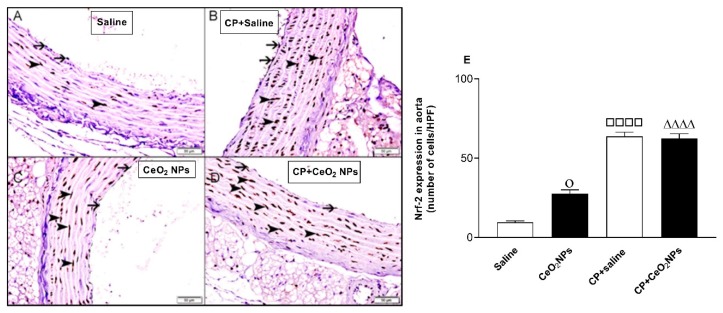
(**A**–**E**): Immunohistochemical analysis of the aortic tissue sections for the detection of nuclear factor erythroid-derived 2-like 2 (Nrf2) in rats intratracheally instilled with either saline (control) or cerium oxide nanoparticles (CeO_2_ NPs) with or without cisplatin (CP) administration. (**A**) Representative section of the aorta of saline-treated rats showing normal aorta with nuclear staining of a few endocardial cells and smooth muscles with Nrf2. (**B**) Representative section of the aorta of rats treated with CP and intratracheally instilled with saline showing increased number of nuclei of smooth muscles and endocardial cells stained with Nrf2. (**C**) Representative section of the aorta of CeO_2_ NPs-treated rats showing increased number of nuclei of smooth muscles and endocardial cells stained with Nrf2. (**D**) Representative section of the aorta of rats treated with CP and intratracheally instilled with CeO_2_ NPs showing increased number of nuclei of smooth muscles and endocardial cells stained with Nrf2. (**E**) Quantification of the numbers of nuclei of smooth muscles and endocardial cells stained with Nrf2 per high power field (HPF) in rats intratracheally instilled with either saline (*n* = 6) or CeO_2_ NPs (*n* = 6) with (*n* = 6) or without (*n* = 6) CP administration. ^ΔΔΔΔ^ *P* < 0.0001 compared with CeO_2_ NPs-treated group. ^□□□□^ *P* < 0.0001 compared with saline-treated group. ^ο^ *P* < 0.05 compared with saline-treated group. Data are mean ± SEM.

At the time point investigated, the study of Nrf2 expression, a transcription factor which plays an important role in the instigation of antioxidant enzymes to respond to oxidative stress, in aorta of saline-instilled control rats showed normal aorta with Nrf2 nuclear staining of a few endocardial cells and smooth muscles (Figure 3A,E). The number of nuclei of smooth muscles and endocardial cells stained with Nrf2 in CP + saline group (Figure 3B,E) was significantly elevated compared with that in the saline group (Figure 3A,E). Likewise, the numbers of nuclei of smooth muscles and endocardial cells stained with Nrf2 in CeO_2_ NPs (Figure 3C,E) were significantly increased compared with the saline group (Figure 3A,E). Moreover, the concomitant administration of CP and CeO_2_ NPs (Figure 3D,E) further increased the number of nuclei of smooth muscles and endocardial cells stained with Nrf2 compared with CeO_2_ NPs group (Figure 3C,E).

### 3.3. Total NO Concentration in Aortic Homogenates

**Figure 4:**
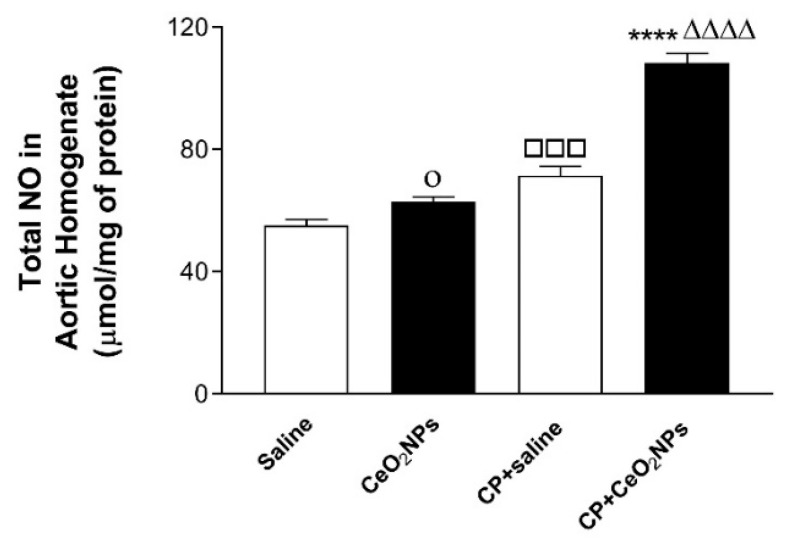
Total nitric oxide (NO) concentration in aortic homogenates of rats intratracheally instilled with either saline (*n* = 5) or CeO2 NPs (*n* = 5) with (*n* = 5) or without (*n* = 5) CP administration. **** *P* < 0.0001 compared with CP + saline-treated group. ^ΔΔΔΔ^ *P* < 0.0001 compared with CeO_2_ NPs-treated group. ^□□□^ *P* < 0.001 compared with saline-treated group. ^ο^ *P* < 0.05 compared with saline-treated group. Data are mean ± SEM.

The concentration of total NO in aortic homogenates was significantly increased 24 h after i.t. installation of CeO_2_ NPs compared with saline (*P* < 0.05), and in CP + saline versus saline (*P* < 0.001). Moreover, the total NO concentration was significantly elevated by the combination of CP and CeO_2_ NPs compared with both CP + saline (*P* < 0.0001) and CeO_2_ NPs (*P* < 0.0001) groups (Figure 4).

### 3.4. LPO Concentrations in Aortic Homogenates

**Figure 5:**
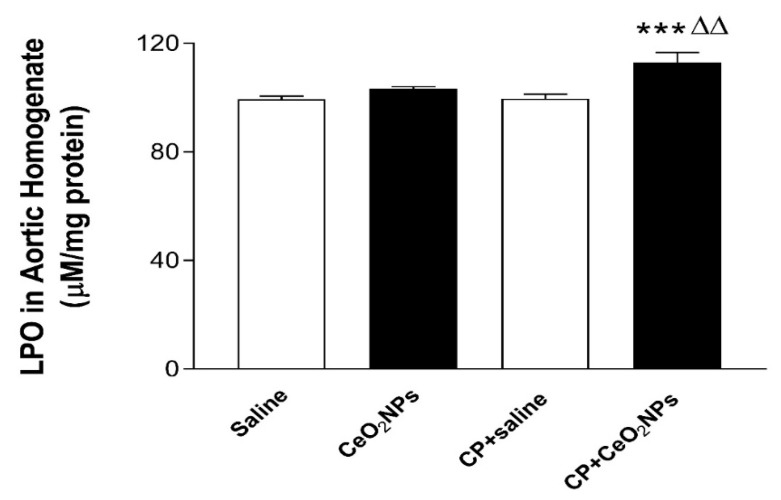
Aortic homogenates concentration of lipid peroxidation (LPO) in rats intratracheally instilled with either saline (control, *n* = 8) or cerium oxide nanoparticles (CeO_2_ NPs, *n* = 7) with (*n* = 8) or without (*n* = 7) cisplatin (CP) administration. *** *P* < 0.001 compared with CP + saline-treated group. ^ΔΔ^ *P* < 0.01 compared with CeO_2_ NPs-treated group. Data are mean ± SEM.

The concentration of LPO, a marker of lipid peroxidation, following pulmonary exposure to either saline or CeO_2_ NPs with or without CP administration are shown in Figure 5. At the time point investigated, the concomitant treatment with CP and CeO_2_ NPs induced a significant increase in the concentration of LPO in aortic homogenates compared with both CP + saline (*P* < 0.001) and CeO_2_ NPs (*P* < 0.01) groups.

### 3.5. 8-OH-dG Concentrations in Aortic Homogenates

**Figure 6:**
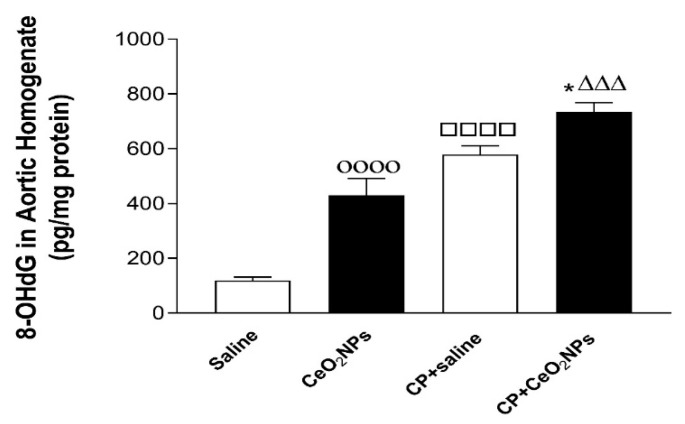
Aortic homogenates concentration of 8-hydroxy-2-deoxyguanosine (8-OH-dG) in rats intratracheally instilled with either saline (*n* = 6) or CeO2 NPs (*n* = 6) with (*n* = 5) or without (*n* = 6) CP administration. * *P* < 0.05 compared with CP + saline-treated group. ^ΔΔΔ^ *P* < 0.001 compared with CeO_2_ NPs-treated group. ^□□□□^ *P* < 0.0001 compared with saline-treated group. ^οοοο^ *P* < 0.0001 compared with saline-treated group. Data are mean ± SEM.

The concentration of 8-OH-dG, a marker of oxidative DNA damage, in aortic homogenates was significantly increased 24 h following pulmonary administration to CeO_2_ NPs compared with saline group (*P* < 0.0001), and in CP + saline versus saline (*P* < 0.0001). Moreover, the concentration of 8-OH-dG was significantly increased by the combination of CP and CeO_2_ NPs compared with both CP + saline (*P* < 0.05) and CeO_2_ NPs (*P* < 0.001) groups (Figure 6).

### 3.6. IL-6 and TNFα Concentrations in Aortic Homogenates

**Figure 7:**
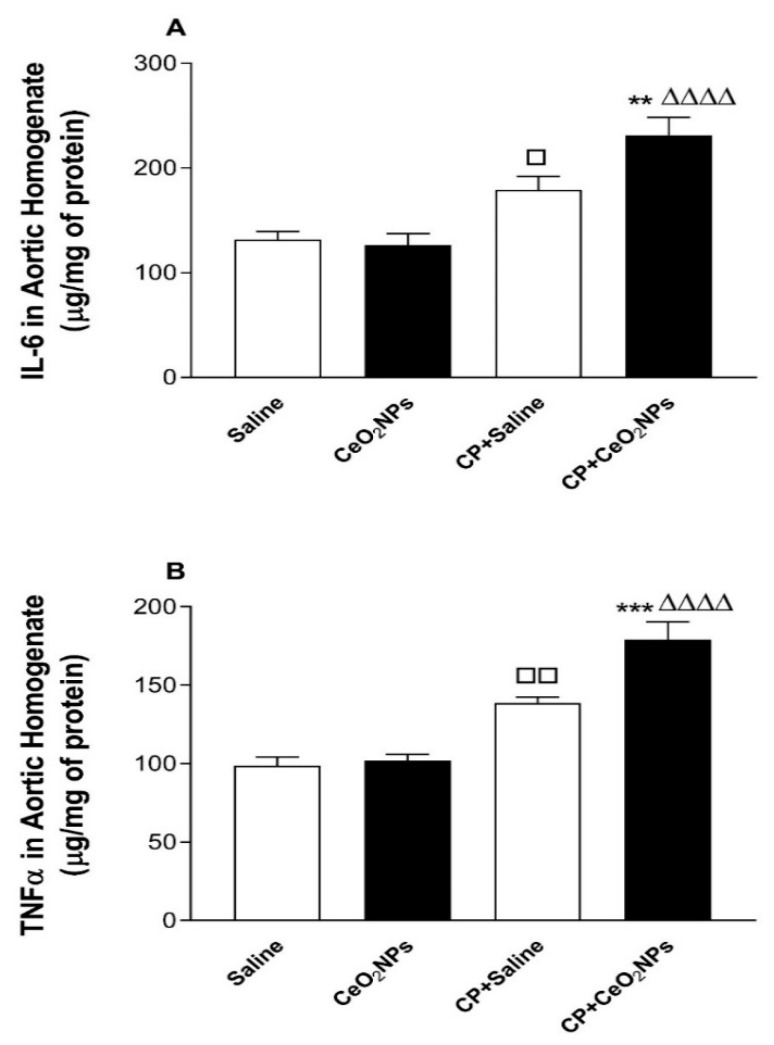
Aortic homogenates concentration of interleukin-6 (IL-6, (**A**)) in rats intratracheally instilled with either saline (control, *n* = 8) or cerium oxide nanoparticles (CeO_2_ NPs, *n* = 8) with (*n* = 8) or without (*n* = 8) cisplatin (CP) administration. Tumour necrosis factor-α (TNFα, (**B**)) concentration in aortic homogenates of rats intratracheally instilled with either saline (n = 8) or CeO_2_ NPs (*n* = 8) with (*n* = 8) or without (*n* = 8) CP administration. ** *P* < 0.01 and *** *P* < 0.001 compared with CP + saline-treated group. ^ΔΔΔΔ^ *P* < 0.0001 compared with CeO_2_ NPs-treated group. ^□^ *P* < 0.05 and ^□□^ *P* < 0.01 compared with saline-treated group. Data are mean ± SEM.

Figure 7 illustrates the effects of pulmonary exposure to either saline or CeO_2_ NPs with or without CP administration on the aortic homogenate concentrations of proinflammatory cytokines, IL-6 and TNFα. Figure 7A shows that, at the time point assessed, the concentration of IL-6 was significantly increased in CP + saline group compared with saline group (*P* < 0.05), and in CP + CeO_2_ NPs compared with either CP + saline (*P* < 0.01) or CeO_2_ NPs (*P* < 0.0001) group. Similarly, as depicted in Figure 7B, the concentration of TNFα in aortic homogenates was significantly augmented in CP + saline group compared with saline group (*P* < 0.01), and in CP + CeO_2_ NPs compared with either CP + saline (*P* < 0.001) or CeO_2_ NPs (*P* < 0.0001) group.

### 3.7. Aortic DNA Damage

**Figure 8:**
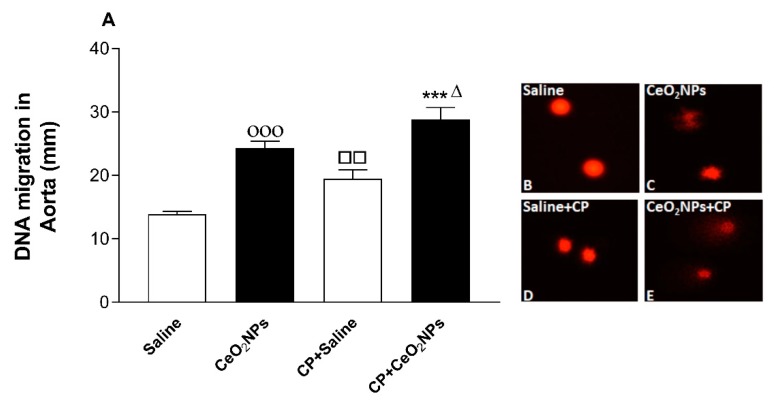
DNA migration (mm) in the aortic tissues evaluated by Comet assay in rats intratracheally instilled with either saline (control, *n* = 5) or cerium oxide nanoparticles (CeO_2_ NPs, *n* = 5) with (*n* = 5) or without (*n* = 5) cisplatin (CP) administration. *** *P* < 0.001 compared with CP + saline-treated group. ^Δ^ *P* < 0.05 compared with CeO_2_ NPs-treated group. ^□□^ *P* < 0.01 compared with saline-treated group. ^οοο^ *P* < 0.001 compared with saline-treated group. Data are mean ± SEM. Images illustrating the quantification of DNA migration by the Comet assay under alkaline conditions in saline, CeO_2_ NPs with or without CP administration.

Figure 8 depicts the effect of treatments on the aortic DNA damage assessed by the Comet assay. Compared with saline-exposed group, 24 h following lung exposure to CeO_2_ NPs, there was a significant augmentation in aortic DNA injury (*P* < 0.001). Likewise, the latter was significantly increased in the CP + saline group compared with the saline group (*P* < 0.01). Furthermore, the degree of aortic DNA damage was further elevated in the CP + CeO_2_ NPs group compared with either CP + saline (*P* < 0.001) or CeO_2_ NPs alone (*P* < 0.05).

## 4. Discussion

In this study, we provide experimental evidence that the vascular pathophysiological effects of pulmonary exposure to CeO_2_ NPs are potentiated in a rat model of vascular injury. Our study demonstrates that the combination of CP and CeO_2_ NPs exacerbates the aortic oxidative stress, Nrf2 expression, inflammation and DNA damage.

It has been demonstrated that inhalation of CeO_2_ NPs causes lung toxicity, crosses the air–blood barrier, and reaches extrapulmonary organs [9]. We have previously reported that i.t. instillation of CeO_2_ NPs in mice induces thrombotic complications and causes toxicity in various organs [8,31]. Moreover, it has been reported that pulmonary exposure to CeO_2_ NPs causes vascular dysfunction and induces exacerbation of myocardial ischemia/reperfusion injury in mice [32]. It is well-established that the use of the anti-cancer drug CP is associated with several adverse effects, especially nephrotoxicity and vascular injury, and hence it is widely used experimentally in rats to induce kidney, cardiac and vascular injury [12,13,14,17,20]. However, as far as we are aware, no study has investigated systematically the oxidative stress, Nrf2 expression, inflammation and DNA damage in aortic tissue of rats treated CP to induce vascular injury and i.t. instilled with CeO_2_ NPs. The i.t. instillation technique used in the present study is simpler than inhalation, thus permitting the administration of a range of doses to the lung in a short-time [1,3,4]. Additionally, the latter technique delivers more accurate dosing, taking into consideration that rats are nose breathers that filter most inhaled particles [1,3,4] The experimental approach used here consisting of assessing the effects of pulmonary exposure to CeO_2_ NPs in a rat model of vascular injury induced by CP is relevant because it is well-established that patients with compromised vascular homeostasis have increased susceptibility to the effects of particulate air pollution [3,10,11]. We have recently demonstrated that the presence of CeO_2_ NPs (1 mg/kg) in the lung exacerbated the renal and lung effects of CP-induced nephrotoxicity in rats [17]. In this study, we used the same dose of CeO_2_ NPs and experimental protocol, and focused our work on the mechanism of action in the aortic tissue. Oxidative stress has been acknowledged as playing a key role in the development of vascular damage [33]. Here we measured in aortic homogenates GSH, a free radical scavenger, and two major antioxidant enzymes, namely CAT and SOD. Our data show that the concentrations of GSH and the activity of CAT were augmented in rats administered with CeO_2_ NPs and CP compared with either CP + saline or CeO_2_ NPs. The augmentation of GSH and CAT suggests that the increase in oxidative stress was associated with an elevation of antioxidant capacity, indicating the occurrence of an adaptive responses that counterweight the potentially detrimental activity of oxygen radicals and reducing further oxidant-mediated aortic damage [8,26]. On the other hand, we found that SOD activity was significantly reduced in CeO_2_ NPs + CP compared with both CP + saline and CeO2 NPs, suggesting a consumption of this antioxidant during the breakdown of free radicals [8,26]. Nrf2 is a transcription factor which is triggered by reactive oxygen species in the vasculature causing the upregulation of several antioxidant genes [34,35]. Proatherogenic conditions induced by metabolic diseases or cigarette smoking have been shown to increase the production of reactive oxygen species in arteries which in turn trigger adaptive mechanisms involving the induction of Nrf2 [34,35]. Here, we show that the expression of Nrf2 by the nuclei of smooth muscles and endocardial cells was significantly augmented in CeO_2_ NPs versus saline and in CP + saline versus saline. Moreover, Nrf2 expression was significantly increased in CP + CeO_2_ NPs versus CeO_2_ NPs. However, unlike the measured antioxidants which were potentiated in CP + CeO_2_ NPs versus CP + saline, the levels of Nrf2 expression in the aorta was comparable in these two groups. The reason for this finding is uncertain, and additional work is required to clarify this point. Moreover, in the present study, we assessed the total NO; LPO, which is a marker of lipid peroxidation; and 8-OH-dG, a marker of oxidative stress to DNA. Our data show a significant elevation of the total NO in aortic homogenates of rats exposed to CeO_2_ NPs and CP compared with both CP + saline and CeO_2_ NPs. Moreover, the concentrations of LPO and 8-OH-dG were significantly increased in rats treated with CeO_2_ NPs and CP compared with both CP + saline and CeO_2_ NPs. The latter findings indicate the occurrence of lipid peroxidation and oxidative DNA damage. Similar to the markers of oxidative stress, the concentrations of the proinflammatory cytokines IL-6 and TNFα were also significantly potentiated by the combination of CP and CeO_2_ NPs versus either CP + saline or CeO_2_ NPs. These results indicate that the aortic inflammation and oxidative stress responses to pulmonary exposure to CeO_2_ NPs are aggravated in rats with compromised vascular homeostasis. Inflammation and proxidant–antioxidant disproportion could exert a substantial role in the occurrence and progression of cardiovascular dysfunction [36]. Moreover, it is well-established that the increase of pro-inflammatory cytokines and reactive oxygen species can induce damage of biomolecules, comprising DNA [36]. The occurrence of DNA damage following exposure to CeO_2_ NPs has been previously reported in vitro using human skin melanoma cells, human dermal fibroblasts, mouse spermatozoa and oocytes, and in vivo in various organs of mouse after i.t. instillation [8,37,38,39,40]. Our data show the occurrence of aortic DNA damage evidenced by COMET assay. In fact, DNA damage in the aorta was significantly increased in CeO_2_ NPs compared with saline, and in CP + saline versus saline, and these effects were significantly aggravated by the combination of CP and CeO_2_ NPs. This effect has, as far as we know, not been reported in aorta before. It has been recently demonstrated that the i.t. administration of CeO_2_ NPs in CP-treated rats exacerbated DNA damage in the kidney and lung [17].

## 5. Conclusions

We conclude that pulmonary exposure to CeO_2_ NPs induces impairment of vascular homeostasis in animal model of vascular injury through mechanisms involving oxidative stress, Nrf2 expression, inflammation and DNA damage. Our study illustrates the relevance of performing exhaustive assessment of the toxicity of CeO_2_ NPs in animal models of human diseases which simulate vulnerable patients with pre-existing vascular diseases.
